# Local- and regional-scale air pollution modelling (PM_10_) and exposure assessment for pregnancy trimesters, infancy, and childhood to age 15 years: Avon Longitudinal Study of Parents And Children (ALSPAC)

**DOI:** 10.1016/j.envint.2018.01.017

**Published:** 2018-04

**Authors:** John Gulliver, Paul Elliott, John Henderson, Anna L. Hansell, Danielle Vienneau, Yutong Cai, Adrienne McCrea, Kevin Garwood, Andy Boyd, Lucy Neal, Paul Agnew, Daniela Fecht, David Briggs, Kees de Hoogh

**Affiliations:** a MRC-PHE Centre for Environment and Health, Department of Epidemiology and Biostatistics, Imperial College London, London, United Kingdom; b UK Small Area Health Statistics Unit (SAHSU), Department of Epidemiology and Biostatistics, Imperial College London, London, United Kingdom; c Population Health Sciences, Bristol Medical School, Bristol, United Kingdom; d Swiss Tropical and Public Health Institute, Basel, Switzerland; e University of Basel, Basel, Switzerland; f Met Office, Exeter, United Kingdom

**Keywords:** ALSPAC, Air pollution, Dispersion modelling, Exposure assessment, Mother-child, PM_10_

## Abstract

We established air pollution modelling to study particle (PM_10_) exposures during pregnancy and infancy (1990–1993) through childhood and adolescence up to age ~15 years (1991–2008) for the Avon Longitudinal Study of Parents And Children (ALSPAC) birth cohort. For pregnancy trimesters and infancy (birth to 6 months; 7 to 12 months) we used local (ADMS-Urban) and regional/long-range (NAME-III) air pollution models, with a model constant for local, non-anthropogenic sources. For longer exposure periods (annually and the average of birth to age ~8 and to age ~15 years to coincide with relevant follow-up clinics) we assessed spatial contrasts in local sources of PM_10_ with a yearly-varying concentration for all background sources. We modelled PM_10_ (μg/m^3^) for 36,986 address locations over 19 years and then accounted for changes in address in calculating exposures for different periods: trimesters/infancy (n = 11,929); each year of life to age ~15 (n = 10,383). Intra-subject exposure contrasts were largest between pregnancy trimesters (5^th^ to 95^th^ centile: 24.4–37.3 μg/m^3^) and mostly related to temporal variability in regional/long-range PM_10_. PM_10_ exposures fell on average by 11.6 μg/m^3^ from first year of life (mean concentration = 31.2 μg/m^3^) to age ~15 (mean = 19.6 μg/m^3^), and 5.4 μg/m^3^ between follow-up clinics (age ~8 to age ~15). Spatial contrasts in 8-year average PM_10_ exposures (5^th^ to 95^th^ centile) were relatively low: 25.4–30.0 μg/m^3^ to age ~8 years and 20.7–23.9 μg/m^3^ from age ~8 to age ~15 years. The contribution of local sources to total PM_10_ was 18.5%–19.5% during pregnancy and infancy, and 14.4%–17.0% for periods leading up to follow-up clinics. Main roads within the study area contributed on average ~3.0% to total PM_10_ exposures in all periods; 9.5% of address locations were within 50 m of a main road. Exposure estimates will be used in a number of planned epidemiological studies.

## Highlights

• PM_10_ exposure assessment for a large birth cohort (pregnancy, infancy, childhood)
• Separate exposures for local (main roads, other) and regional/long-range sources.
• Primary PM_10_ from local major roads on average was 3% of total PM_10_ exposures.
• Largest contrasts for pregnancy trimesters (5^th^ to 95^th^ centile: 24.4–37.3 μg/m^3^)
• Mean PM_10_ exposures fell from 31.2 μg/m^3^ age ~1 to 19.6 μg/m^3^ age ~15 years.

## Graphical abstract

**Figure:**
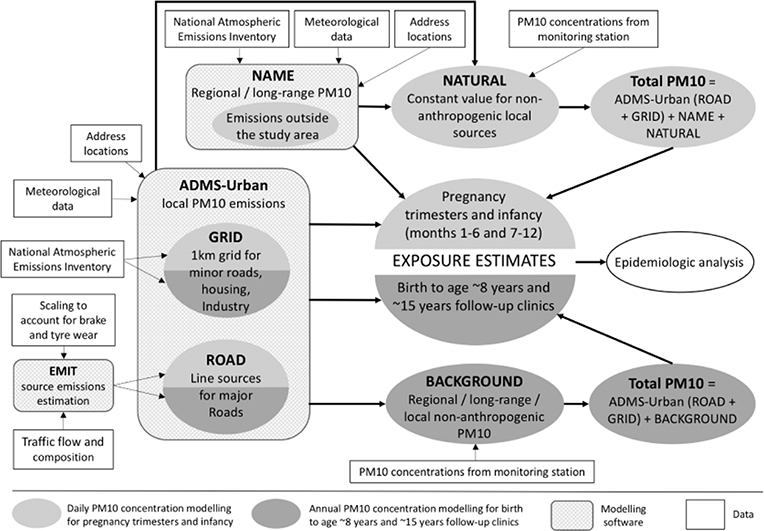

## 1 Introduction

Relationships of air pollution exposure and children's respiratory health, including lung function and asthma symptoms, have been reported in a growing number of studies, across which different exposure assessment approaches and time windows were used. Some reported associations of adverse respiratory outcomes with air pollution exposures at birth (Gehring et al., 2015), infancy (Schultz et al., 2016), pre-school age (Brunst et al., 2015), and mid-childhood (Gauderman et al., 2015; Rice et al., 2016), while others did not observe associations in any of these time windows (Fuertes et al., 2013; Fuertes et al., 2015; Molter et al., 2015). Little is known about in utero exposure and its lasting influences on childhood respiratory outcomes. Only recently have links between trimester-specific exposures and childhood asthma (Hsu et al., 2015; Sbihi et al., 2016) or lung function levels (Morales et al., 2015) been reported.

The Avon Longitudinal Study of Parents And Children (ALSPAC), established in 1990, is a prospective birth cohort recruited during pregnancy from a defined geographical population in the south west of England that collected data on the health and development of children through to adolescence (Boyd et al., 2013). As part of a study on “Effects of early life exposure to particulates on respiratory health through childhood and adolescence” we aimed to 1) determine particles < 10 μm in diameter (PM_10_) exposures during the critical periods of lung development from the fetal period (by pregnancy trimester) (Morales et al., 2015; Mortimer et al., 2008) through early (months 1 to 6) and late (months 7 to 12) infancy, and 2) long-term (i.e. averaging periods of 1-year or more) exposures up to age ~8 and ~15 years when assessments of asthma, lung function and bronchial responsiveness, and sensitization to allergens were made at follow-up clinics. Using the residential address history of participants, we endeavored to use air pollution models to estimate time-weighted PM_10_ exposures for each of the above periods.

In the ALSPAC study area there was no more than one fixed-site PM_10_ monitoring site operating at any time during the study period. There was no PM_10_ monitoring during all periods of pregnancy (1990–1992) and only partial coverage for periods of infancy for some of the cohort (1993-). We therefore used dispersion modelling (Atkinson et al., 2015; Baiz et al., 2012; Charpin et al., 2009; Bellander et al., 2001; Butland et al., 2017; de Hoogh et al., 2014; Gong et al., 2017; Hansen et al., 2016; Korek et al., 2015; Nordling et al., 2008; Pirani et al., 2014; Van den Hooven et al., 2012; Schultz et al., 2016; Vinceti et al., 2016) to estimate population exposure. There are, however, only a few examples of using city/regional scale dispersion modelling for historic sub-annual exposure periods for particles (Baiz et al., 2012; Nordling et al., 2008; Heck et al., 2013; Gong et al., 2017; Rahmalia et al., 2012; Sellier et al., 2014) due to the challenge of developing detailed inventories on sources and emissions of air pollution at different spatial scales (e.g. local, regional, long-range).

This paper describes the development and evaluation of air pollution modelling (PM_10_) and exposure assessment for 1990 to 2008, a time period covering all periods of pregnancy, infancy, and childhood/adolescence in the ALSPAC cohort. We describe the development of dispersion models to estimate PM_10_ concentrations within the study area, and how we evaluated model performance against data collected from one routine measurement site. The magnitude and variability of exposures for total PM_10_ and separately for local and non-local estimates of PM_10_ are described for each period.

## 2 Methods

### 2.1 Cohort and study area

Women were eligible to join ALSPAC if they were due to deliver between the 1^st^ April 1991 and 31^st^ December 1992, while living in a defined catchment area in and around the city of Bristol (described below). The ALSPAC eligible sample includes 20,248 known pregnancies, 20,390 known fetuses resulting in 19,600 live births. ALSPAC initially recruited mothers carrying 14,541 pregnancies (August 1990 to December 1992), with 14,062 subsequent live born infants (due for delivery between April 1991 to December 1992), of which 13,988 survived to the end of the first year of life. All individuals in the original eligible sample remain eligible, and - by the time the index children reached age 18 years - the enrolled cohort had increased to include 15,247 pregnancies and 14,775 live-born infants, of which 14,701 were alive at 1 year of age. ALSPAC has established an exceptionally detailed data and biological resource from pregnancy (see http://www.bris.ac.uk/alspac/researchers/data-access/data-dictionary/).

**Fig. 1:**
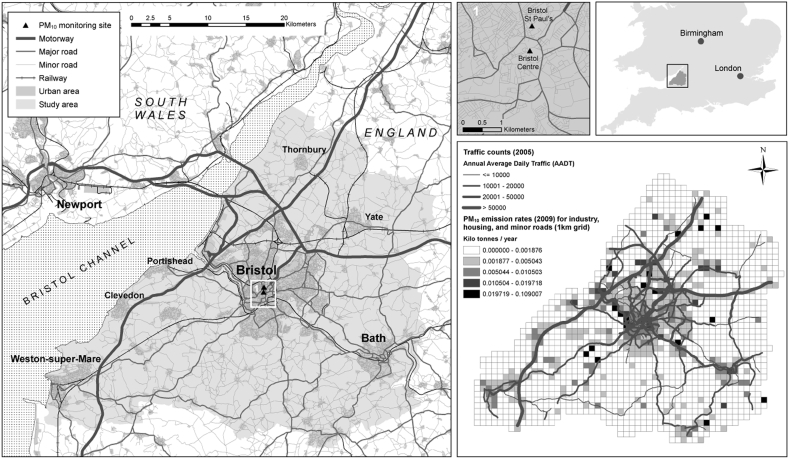
Extent of the study area, location of PM_10_ monitoring sites, and geographical distribution of baseline traffic volumes and emissions rates from industry, housing, and minor roads.

The ALSPAC cohort was recruited from three district health authorities (DHAs) (Bristol & Weston, Fenchay, Southmead) in the south west of England (Boyd et al., 2013) that include the city of Bristol and the towns of Clevedon, Portishead, Thornbury, and Weston-Super-Mare. Our study area (Fig. 1) is the defunct county (replaced by four “unitary authorities” in 1996) of Avon (1333 km^2^) including the ALSPAC recruitment DHAs, and also the city of Bath and a predominantly rural area (Wansdyke) to the south of Bristol to retain people who moved out of the recruitment DHAs to neighboring areas included in follow-up clinics. Our study area, however, fails to capture participants who moved further distances from the original ALSPAC catchment area. In the 1991 UK Census (ONS: https://www.ons.gov.uk) the population of Avon was 957,981 and the city of Bristol had a population of 376,146. By 2008 the population of Avon (i.e. the four unitary authorities that replaced Avon) was 1,047,091 and Bristol had a population of 415,122. The coastal area to the West of Bristol includes the port of Avonmouth, historically an area of heavy industry, including several chemical plants and gas-fired power stations. A number of major road arteries (e.g. M32, M4, M5 motorways) run through the study area.

[©Crown copyright/Mastermap 2017. Ordnance Survey/Edina supplied service.]

### 2.2 Measured PM_10_ concentrations (1993–2008)

Prior to the early 1990s air pollution monitoring in the UK was limited to non-automatic networks (http://uk-air.defra.gov.uk/networks/) including daily concentrations from the ‘Smoke and Sulphur Dioxide network’, and annual estimates of NO_2_ from the ‘Diffusion Tube Network’ (Bush et al., 2001). Automatic, hourly measurements of PM_10_ and other pollutants started in the UK in 1992, and are currently operated by the Department of Environment, Food and Rural Affairs (Defra), as part of the UK Automatic Urban and Rural Network (AURN) of air pollution monitoring sites. For the period of our study, monitoring in the Bristol area varied, but remained limited: from 1990 to 1992 there was no PM_10_ air pollution monitoring in the area; from January 1993 until September 2005 there was a single site called ‘Bristol Centre’, classified as ‘urban centre’; Bristol Centre was relocated about 0.5 km to ‘Bristol St Paul's’ in July 2006 (Fig. 1) and classified as an ‘urban background’ site; since 2008, Bristol St Paul's has recorded both PM_10_ and PM_2.5_. We intended to also model PM_2.5_ exposures but there were not any modelling options or measurements in the study area until 2008. We downloaded hourly concentrations of PM_10_ from the AURN data archive (http://uk-air.defra.gov.uk/data/) for the Bristol Centre and Bristol St Paul's monitoring sites. We used the hourly data to calculate daily, monthly, and annual PM_10_ concentrations.

### 2.3 Modelling strategy

**Fig. 2:**
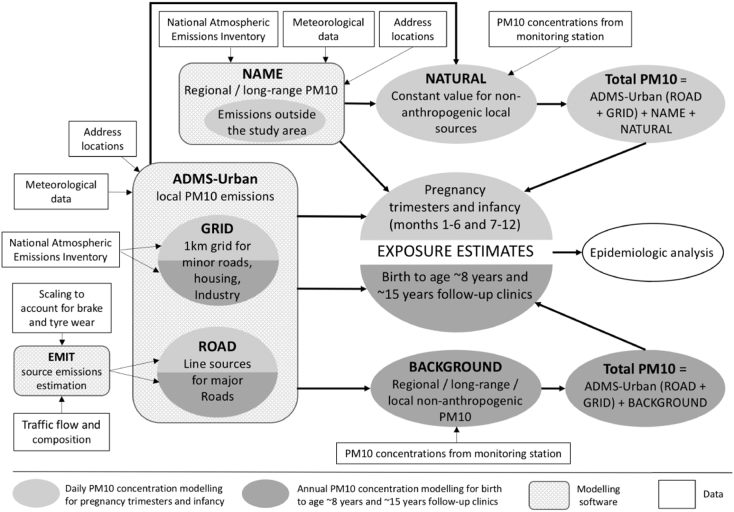
Overview of data and models used for estimating PM_10_ exposures.

We used dispersion models to predict PM_10_ exposures at residential address locations. An overview of the data and models used to estimate exposures is shown in Fig. 2.

#### 2.3.1 Pregnancy and infancy exposures

We required exposures for each pregnancy trimester (T1, T2, T3), and for early (1–6 months) and late (7–12 months) infancy (EI and LI). We applied dispersion models, operating on different geographical scales to account for both spatial and temporal variations in daily PM_10_, and then we averaged daily PM_10_ concentrations for each exposure period on an individual basis. ADMS-Urban was used for local sources (i.e. within the study area), and NAME-III for regional/long-range sources (i.e. outside the study area).

ADMS-Urban (Carruthers et al., 1994) is a proprietary, “advanced” dispersion model designed to represent emissions from individual sources (e.g. lines representing roads, and industrial point and area sources) at city/region scale. Aggregated and diffuse sources can be modelled on a continuous regular grid. ADMS-Urban has been used for local air quality management and exposure studies (Baiz et al., 2012; Hampel et al., 2011; Pirani et al., 2014; Rahmalia et al., 2012; Sellier et al., 2014). We did not have historic emissions information for specific industrial and domestic sources or minor roads, so we modelled combined emission rates of PM_10_ from these sources using information available on a 1 km grid (GRID) from the National Atmospheric Emissions Inventory (NAEI, 2009) (http://naei.defra.gov.uk/data/mapping), and modelled emissions from each main road as a line source (ROAD). Main roads typically have annual average daily traffic (AADT) >10,000, but some roads with 5000–10,000 AADT are also included because they are thought to have roadside levels of air pollution comparable to roads with >10,000 AADT, such as congested areas or street canyons which may have poor ventilation.

NAME-III (Numerical Atmospheric-dispersion Modelling Environment) (Jones et al., 2007), hereafter referred to as NAME, is a sophisticated, off-line, Lagrangian dispersion and air quality model developed by the Met Office. NAME has a full tropospheric chemistry and aerosol modelling capability. It uses full 3D meteorological fields, normally derived from the Met Office's Numerical Weather Prediction (NWP) model ‘MetUM’. Information on source emissions in NAME was from the NAEI for the UK, but up-scaled to a grid resolution of ~17 km for model runs, and from the European Monitoring and Evaluation Programme (EMEP) (http://www.emep.int) on a 50 km grid for north-west Europe (Belgium, Denmark, France, Germany, Luxembourg, the Netherlands, Norway, Sweden), using data from representative annual totals going back to 1990. In the present study, due to our requirement for predictions back to 1990, meteorological data were used from a meteorological reanalysis. To provide estimates of regional, secondary and long-range transported pollution, emissions of primary particulate matter in the study area were removed from NAME to avoid double-counting the pollution estimates derived from ADMS-Urban.

We also included a constant in the model (NATURAL) to account for local non-anthropogenic sources of PM_10_ (e.g. wind-blown soil and other crustal matter). Thus, total short-term PM_10_ (ST-PM10_TOTAL_), Eq. (1), is the sum of the average of each model component by exposure period (i.e. trimester, early/late infancy).

(1) $$\mathrm{ST}-\mathrm{PM}10_{\text{TOTAL}}=\text{ADMS}-\text{Urban}\;\left(\text{ROAD}+\text{GRID}\right)+\text{NAME}+\text{NATURAL}$$

#### 2.3.2 Exposures through childhood and adolescence

For periods relating to childhood and adolescence we were interested in summarizing average long-term (i.e. over several years) exposures, so we modelled annual average concentrations and then averaged those for different periods of interest (e.g. from birth to follow-ups clinics at age ~8 years and ~15 years). Long-term (i.e. over several years) exposures relate to spatial contrasts in PM_10_ concentrations so we used ADMS-Urban, again separately for main roads (ROAD) and other sources (GRID). We did not use NAME - a model used mostly to describe temporal rather than spatial contrasts for our study area - so we included a variable called BACKGROUND to represent local non-anthropogenic particles and regional and long-range particles from both anthropogenic and non-anthropogenic sources. Total long-term PM_10_ (LT-PM10_TOTAL_) is thus calculated as Eq. (2).

(2) $$\mathrm{LT}-\mathrm{PM}10_{\text{TOTAL}}=\text{ADMS}-\text{Urban}\;\left(\text{ROAD}+\text{GRID}\right)+\text{BACKGROUND}$$

### 2.4 Modelling local sources with ADMS-Urban

#### 2.4.1 PM_10_ emissions from main roads (ROAD)

Information on annual average daily traffic (AADT) flows (i.e. number of vehicles per road section) and composition (i.e. the proportion of light and heavy goods vehicles), and speeds (km h^−1^), were provided for main roads (n = 419; 27 motorway sections and 392 other main road sections) by Bristol City Council (BCC) for 2005 for the city of Bristol and some of the surrounding area. This was supplemented with data obtained from the UK Department of Transport (https://www.dft.gov.uk/traffic-counts/) for other motorways and main road sections (n = 1319). This provided a total of 1738 road sections with traffic information. Traffic data were linked to the geography of the road network (OS MasterMap® Integrated Transport Network™) (Fig. 1). Historical traffic data are scarce, but we had access to paper records on traffic counts undertaken in 1993 by BCC for a limited number (n = 25) of road sections. We used the strong relationship (R^2^ = 0.93; p = 0.000) of concomitant traffic counts from 1993 and 2005 to impute a 1993 traffic data set for all roads. Traffic counts for other years (1990–1992; 1994–2005; 2007–2008) were imputed by linear extrapolation, following the method by Keuken et al. (2011), based on the average difference between 1993 and 2005 traffic flows. We had data on traffic speeds by road link for 2005 and assumed these to be constant over the study period.

Tail-pipe emission rates (g km^−1^) were calculated from traffic flows and speeds for each road section using the Emissions Inventory Toolkit (EMIT), the partner software to ADMS-Urban. A substantial proportion of traffic-related PM_10_ also comes from brake/tyre wear and road abrasion, which could not be calculated using EMIT. We therefore used data in the NAEI (http://naei.beis.gov.uk/data/data-selector) to calculate year-specific ratios of tail-pipe to total traffic-related emissions in order to scale estimated tail-pipe emissions from EMIT to total emissions (tail-pipe, brake/tyre wear, road abrasion) for each road section by year. National emissions of PM_10_ by source sector are shown in Fig. S1 (Supporting information). In 1990, 11.0% and 4.2% of total national PM_10_ emissions were from vehicle tail-pipe and non-tailpipe, respectively. In 2008, 11.0% and 12.0% of total national PM_10_ emissions were from tail-pipe and non-tailpipe, respectively. Although total PM_10_ emissions have reduced (by 58%) over the study period, the proportion of PM_10_ related to road traffic has increased. We included road width for all roads, and building heights for continuous street canyons, in ADMS-Urban; it was not possible to model the effects of partial canyons.

#### 2.4.2 PM_10_ emissions from industry, housing and minor roads (GRID)

We used data from the NAEI (http://naei.beis.gov.uk/data/data-selector) to calculate emissions from other sources (industry, housing, minor roads) within the study area. The NAEI has total emissions of PM_10_ on a 1 × 1 km grid which includes emissions from main roads. We downloaded this data for the whole of Great Britain and extracted the grid sources for the study area using ArcGIS (ESRI Ltd., Redlands, California, USA). We intersected the geography of main roads with the 1 × 1 km grid, summed emission rates from main roads within each 1 km × 1 km grid square, and then subtracted these values from total PM_10_ emissions within each square to ensure there was no double counting of emission sources between GRID and ROAD. We then used information from the NAEI on changes in annual total emissions in Great Britain (Fig. S1, Supporting information) to scale baseline 2009 emissions across the 1 km × 1 km grid for each year in our study period (1990–2008). The geography of road and grid sources, each with associated emission rates of PM_10_ (Fig. 1) for each year, were subsequently imported into ADMS-Urban.

#### 2.4.3 Meteorological data

Meteorological variables for the ADMS-Urban modelling were obtained from British Atmospheric Data Centre (www.badc.ac.uk). We used data from the site at Glastonbury (~40 km South of Bristol) because, although it was not the nearest site to the study area, it had continuous coverage of data for our study period. We constructed meteorological data (i.e. ‘MET’) files for each year to cover the period 1990–2008. Each MET file contained hourly values of wind speed, wind direction, cloud cover, and temperature. These data were used in ADMS-Urban to simulate boundary-layer atmospheric conditions and to define the pollutant dispersion parameters.

#### 2.4.4 ADMS-Urban model runs

We used ADMS-Urban version 2.3 (interface 2.26) which allows a maximum of 1500 road sources, 3000 grid sources, and 10,000 receptors (i.e. address point locations) per model run. Our study area included 1738 road sources, 1542 grid sources (i.e. 1 km × 1 km squares), and 36,986 receptors, so we had to separate the data over multiple model runs. We summed ROAD and GRID concentrations from the different model runs applied to each address location to give hourly concentrations of PM_10_ for periods relating to pregnancy and infancy, which we subsequently averaged to daily concentrations in SPSS (IBM) version 20, and annual concentrations of PM_10_ for each year from birth to age ~15 years.

### 2.5 Modelling PM_10_ from sources outside the study area with NAME

Data from NAME were used to provide estimates of regional and long-range transported (i.e. derived from sources outside the Avon study area) PM_10_ concentrations. Meteorological data for NAME modelling was taken from the ERA-Interim meteorological reanalysis produced by ECMWF (Berrisford et al., 2011). This provides analysis fields at 6-hourly intervals and at approximately 79 km horizontal spatial resolution. Pollution data for periods relating to pregnancy and infancy (1990–1993) were provided in the form of daily average concentrations of PM_10_ for 14 receptor locations, at a regular spacing of 10 km between points over the study area. We assigned daily PM_10_ concentrations from one of the 14 receptor locations to each address location using the ‘nearest neighbor’ tool in ArcGIS.

### 2.6 Natural sources and background PM_10_ concentrations

NATURAL in ST-PM10_TOTAL_ is the difference between the average measured PM_10_ concentration (Bristol Centre, 315 days in 1993) and the average of the sum of daily modelled concentrations from ADMS-Urban (ROAD + GRID) and NAME. For 1990–1992 (i.e. a period without PM_10_ monitoring in the study area) we assumed NATURAL to be the same as from Bristol Centre for 1993. This yielded a constant value of 12.0 μg/m^3^ to represent NATURAL in all daily estimates of PM_10_ concentrations. BACKGROUND in LT-PM10_TOTAL_, varying by year, is the difference between annual average measured PM_10_ concentrations, at either Bristol Centre (1993–2005) or Bristol St Paul's (2006–2008), and the sum of annual average modelled concentration from ADMS-Urban (ROAD + GRID).

### 2.7 Model evaluation

Model evaluation exercises have been undertaken by the producers of ADMS-Urban (Carruthers et al., 2003) and NAME (Jones et al., 2007). We undertook our own model evaluation in this study to inform the epidemiological studies on the ability of models to rank exposures (i.e. correlation) and the potential for bias in exposure estimates. For the period intersecting ST-PM10_TOTAL_ (1990–1993) there was monitoring at Bristol Centre (Fig. 1) for a period covering 315 days during 1993. We created weekly average PM_10_ concentrations from hourly measurements and compared them with estimates of weekly PM_10_ concentrations from ST-PM10_TOTAL_ (Eq. (1)). In the absence of enough monitoring data to evaluate the model for periods relating to pregnancy trimesters, we chose a week as the averaging time for this evaluation as it is a period shorter than our minimum exposure period (trimester), and also a period of time that relates to the broad-scale changes in meteorology that mostly determine the variability in temporal patterns of regional and background PM_10_ concentrations.

We used the following statistical tests in model evaluation exercises: Spearman's rank correlation coefficient (r), the coefficient of determination (MSE-r^2^) [i.e. 1 − (mean squared error of predictions / variance of observations)], root mean square error (RMSE), the variance of observations (measurements) and predictions (modelling), mean absolute bias (MB), mean percentage bias (MPB), the percentage of values within a factor of two (FAC2), and the regression fit line with 95% confidence intervals (CI). We used Spearman's correlation to reflect the skewed nature of PM_10_ concentrations and also because for exposure assessment we are interested in relative ranking of exposures. These statistical tests were chosen to cover the key elements of characterizing and assessing performance of environmental models as described in Bennett et al. (2013).

### 2.8 Geocoding of address locations

ALSPAC provided us with all residential addresses held in the ALSPAC contact database (n = 45,771). Using a geocoding algorithm developed at Imperial College London, based on Ordnance Survey's AddressBase Plus©, we were able to assign geographical coordinates to 96.2% of addresses (n = 44,028). We restricted our study to children that did not move outside the study area between date of conception and the age 15 years at follow-up. This yielded 36,986 addresses for which we modelled daily air pollution concentrations. We produced the Algorithm for Generating Address-history and Exposures (ALGAE; https://smallareahealthstatisticsunit.github.io/algae/index.html) to clean the address history of the cohort so that we could account for residential mobility in estimating exposures. We were able to geocode at least one address within the study area for 14,027 individuals. We selected only those individuals for each exposure period that following the applications of ALGAE had valid addresses for at least 90% of days in trimesters and infancy, and at least 75% of days within each year of life up to age 15 years. Table S2 (Supporting information) shows the number of individuals retained for each exposure period (n: 11,446–12,775). We included only those individuals who met the thresholds for number of valid days of address history in all periods of pregnancy and infancy (n = 11,929) and separately from birth to age 15 years (n = 10,383).

### 2.9 Exposure assessment

For trimesters and infancy exposure periods, we compiled daily total PM_10_ concentrations (ST-PM10_TOTAL_) and separate PM_10_ concentrations for local (i.e. ROAD and GRID) and regional/long-range (NAME) PM_10_, for all address locations for the period August 1st 1990 (i.e. date of registration of the first pregnancy) to 31st December 1993 (i.e. 1st birthday of the youngest child in the cohort). We then calculated address-time-weighted trimester and infancy exposures from the daily estimates. For each year of life up to age 15 years (i.e. year 16) we calculated annual average values of LT-PM10_TOTAL_, ROAD, and GRID. To approximately align with the age ~8 years and age ~15 years follow-up clinics, during which relevant health outcomes were clinically assessed, we used the yearly LT-PM10_TOTAL_, ROAD, and GRID to calculate address-time-weighted exposures for birth to year 8, and years 9 to 16. We produced descriptive statistics for each exposure period and Spearman's (rho) correlations between different exposure periods and between model components (ROAD, GRID, NAME) as epidemiological studies are interested in determining whether findings related to a particular exposure period or if sources are independent. For the purpose of this assessment we defined correlations as very low (≥ 0.0–0.2), low (>0.2–0.4), moderate (>0.4–0.6), high (>0.6–0.8), and very high (>0.8–1.0).

### 2.10 Ethics and governance

To maintain the confidentiality of participant information, all use of participant address information (which carry a high risk of re-identifying participants) was isolated from the use of participant attribute data, and was conducted within the ALSPAC Data Safe Haven and supervised by ALSPAC staff in Bristol. Ethical approval for ALSPAC was obtained from the ALSPAC Ethics and Law Committee and the Local Research Ethics Committees.

## 3 Results

A time-series of annual and monthly mean concentrations of PM_10_ for the period 1993 to 2008 is shown in Fig. S2. There was an overall decline of 16.5 μg/m^3^ in PM_10_ across the study period. In 2003 there was an unusually warm summer characterized by low wind speeds and a number of “air pollution episodes” (Stedman, 2004), hence the notable elevated annual PM_10_ concentration compared to surrounding years. The decline in PM_10_ concentrations follows the national picture of a decline in emissions of PM_10_ (Fig. S1).

### 3.1 Model evaluation

Table 1 and Fig. S3 show model performance for estimated weekly (n = 37) ST-PM10_TOTAL_ at the Bristol Centre monitoring site (1993). ST-PM10_TOTAL_ performed well in terms of correlation (r = 0.71) and predicting the variance (Var_P_ = 226.9) of measured concentrations of PM_10_ (Var_O_ = 220.0). As shown in Fig. S3, 3 of the weekly estimates are not within a factor of two of measured concentrations. The regression fit line shows that overall the model underestimates measured concentrations (MB = −4.1 μg/m^3^, MPB = 21%) and MSE-r^2^ is 0.41. Fig. S4 shows the proportional contribution of each model component (ROAD, GRID, NAME, NATURAL) to ST-PM10_TOTAL_ for each of the 37 weeks. NAME (i.e. regional/long-range PM_10_) dominates weekly estimates of PM_10_ concentrations with the smallest contribution from local main roads (ROAD).

**Table 1:** Performance statistics from comparing modelled and measured PM_10_ concentrations (μg/m^3^): 37 weekly averages at the Bristol Centre monitoring station (3rd January to 26th December 1993; data not available for 13 weeks during this period).

Number of monitoring sites	Year	Averaging period	N	r^a^	MSE-r^2^	Var_O_	Var_P_	RMSE	MB	MPB	FAC2	Regression line		95% β CI (lower, upper)
												β	Constant	
1	1993	Week	37	0.71^b^	0.41^b^	220.0	226.9	11.4	−4.1	21%	81%	0.70	11.34	0.46, 0.93

MSE-r^2^ is the coefficient of determination; Var_O_ is the variance of the measured values (i.e. observations); Var_P_ is the variance of the modelled values (i.e. predictions); RMSE is the root mean squared error; MB is mean absolute bias; MPB is mean percentage bias; FAC2 is percentage of values within a factor of two.

a r is Spearman's rho.

b p = 0.000.

### 3.2 Exposure assessment

**Fig. 3:**
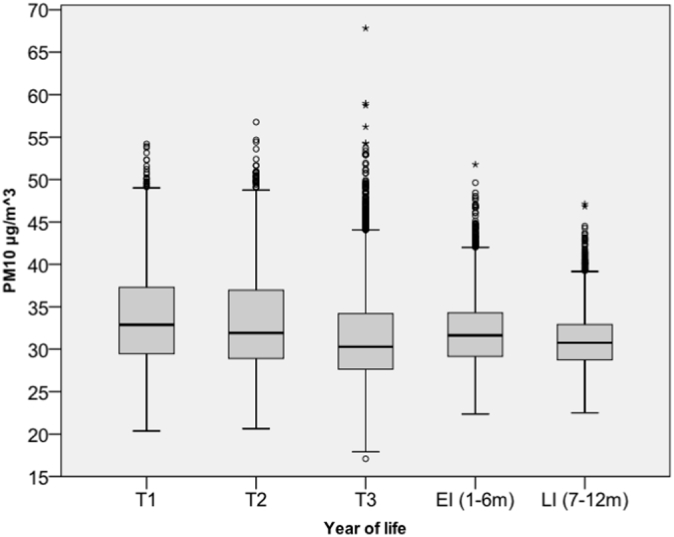
ST-PM10_TOTAL_ exposures (μg/m^3^) for pregnancy trimesters (T1, T2, T2), early infancy (EI; months 1 to 6), and late infancy (months 7 to 12).

Mean ST-PM10_TOTAL_ exposures were 33.5 μg/m^3^ (5^th^ to 95^th^ centile: 25.7–42.6 μg/m^3^) in T1 and declined in each subsequent period to 30.8 μg/m^3^ (5^th^ to 95^th^ centile: 25.9–35.8 μg/m^3^) in LI (Table 2). T3 had the largest number of outliers (circles: >median ± 1.5 × IQR; stars: >median ± 3 × IQR) relating to premature births overlapping with short periods of elevated PM_10_ concentrations (Fig. 3). The contribution to ST-PM10_TOTAL_ was 2.9–3.0% for ROAD, 15.7–16.5% for GRID, 42.2–45.6% for NAME, and 28.9–35.9% for NATURAL. In other words, local sources (ROADS + GRID) on average contributed 18.6–19.5% to ST-PM10_TOTAL_.

**Table 2:** Summary statistics for modelled total [ROAD + GRID + NAME + NATURAL (i.e. a constant value of 12 μg/m^3^)], local major roads (ROADS), local other sources (GRID), and regional/long-range (NAME) PM_10_ exposures (μg/m^3^) for pregnancy trimesters (T1, T2, T3), early infancy (EI, months 1 to 6), and late infancy (LI; months 7 to 12); includes individuals with valid address history in all periods (n = 11,929).

PM_10_ component	Exposure period	Mean	Min	Percentiles					Max	IQR	SD
				5th	25th	50th	75th	95th			
Total^a^	T1	33.6	20.4	25.7	29.5	32.9	37.3	42.6	54.2	7.9	5.3
	T2	33.0	20.7	25.5	28.9	31.9	37.0	42.6	56.8	8.1	5.4
	T3	31.3	17.1	24.4	27.6	30.3	34.2	41.3	67.8	6.6	5.2
	EI	31.9	22.4	26.1	29.2	31.7	34.3	38.4	51.8	5.1	3.8
	LI	30.8	22.5	25.9	28.7	30.7	32.9	35.8	47.1	4.2	3.0
Local main roads (ROAD)	T1	0.96	0.07	0.26	0.48	0.80	1.20	2.29	7.80	0.72	0.70
	T2	0.96	0.10	0.25	0.48	0.80	1.18	2.30	8.37	0.70	0.71
	T3	0.94	0.06	0.25	0.47	0.78	1.17	2.26	7.26	0.70	0.69
	EI	0.94	0.14	0.28	0.46	0.79	1.17	2.19	8.33	0.71	0.68
	LI	0.89	0.13	0.25	0.43	0.75	1.11	2.11	7.17	0.68	0.65
Local other sources (GRID)	T1	5.24	0.78	2.46	3.87	5.10	6.28	8.50	18.36	2.41	1.96
	T2	5.24	0.79	2.46	3.88	5.08	6.29	8.52	19.07	2.41	1.95
	T3	5.16	0.86	2.36	3.81	5.05	6.23	8.45	19.32	2.42	1.93
	EI	5.19	1.05	2.54	3.81	5.14	6.18	8.35	19.47	2.37	1.88
	LI	4.85	1.08	2.37	3.56	4.82	5.78	7.79	17.18	2.22	1.74
Regional/long-range (NAME)^b^	T1	15.24	6.80	8.91	11.87	14.69	19.16	22.72	24.65	7.29	4.20
	T2	14.77	6.80	8.71	11.61	13.38	19.08	22.68	24.65	7.47	4.31
	T3	13.20	3.11	8.38	10.57	12.11	14.93	21.51	49.91	4.36	4.01
	EI	13.72	8.94	10.05	11.24	13.81	15.54	18.33	19.48	4.30	2.58
	LI	13.10	9.01	10.06	11.16	13.40	14.77	15.95	18.62	3.61	1.99

a The Total may not be equal to the sum of other components (ROAD + GRID + NAME + 12) due to rounding.

b Only includes sources located outside the study area (Fig. 1).

**Fig. 4:**
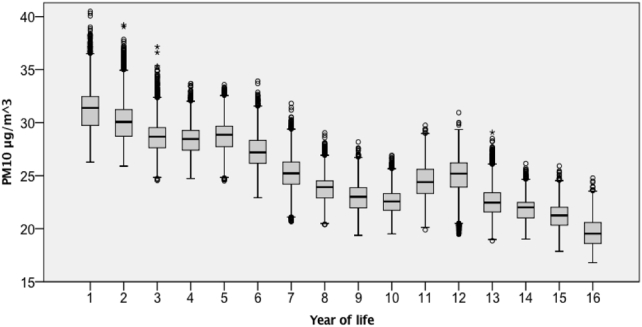
LT-PM10_TOTAL_ exposures (μg/m^3^) for each year of life from birth (i.e. year 1) to age ~15 (i.e. year 16).

There was a substantial reduction in LT-PM10_TOTAL_ exposures by year from year 1 (31.2 μg/m^3^) to year 16 (19.6 μg/m^3^) (Fig. 4, Table S3) that follows the general reduction in measured PM_10_ concentrations across the study period (Fig. S2). There was a notably higher exposure for years 11 and 12 compared to surrounding years (9, 10, 13, 14). This increase was related to a number of air pollution episodes that contributed to higher than normal PM_10_ concentrations for this period of time (i.e. during the 2000s) (Stedman, 2004), which can also be seen in measured PM_10_ concentrations for 2003 (Fig. S2).

There was a 5.4 μg/m^3^ difference in average LT-PM10_TOTAL_ exposures between ages 0–8 years (27.9 μg/m^3^) and ages 9–16 years (22.5 μg/m^3^) (Table 3, Fig. S5). Spatial contrasts in PM_10_ exposures to age ~8 years (5^th^ to 95^th^ centile: 25.4–30.0 μg/m^3^) and age ~15 years (5^th^ to 95^th^ centile: 20.7–23.9 μg/m^3^) were low. Contributions were 2.7–3.1% for ROAD and 11.7–13.9% GRID depending on the time period (Table 3). Local sources contributed 14.4–17.0% to LT-PM10_TOTAL_ with BACKGROUND representing 83.0–85.6% of LT-PM10_TOTAL_.

**Table 3:** Summary statistics for modelled total [ROAD + GRID + BACKGROUND (i.e. a value for each year averaged over each exposure period)], local main roads (ROAD), and local other sources (GRID) PM_10_ exposures (μg/m^3^) for birth to year 8, year 9 to 16, and birth to year 16; includes only those individuals with valid address history in all periods (n = 10,383).

PM_10_ component	Exposure period	Mean	Min	Percentiles					Max	IQR	SD
				5th	25th	50th	75th	95th			
Total^a^	0 to year 8	27.9	24.1	25.4	26.9	28.0	28.8	30.0	34.8	1.9	1.4
	Year 9 to 16	22.5	19.7	20.7	21.7	22.6	23.2	23.9	26.9	1.5	1.0
	0 to year 16	25.2	21.9	23.1	24.4	25.3	26.0	26.9	30.8	1.6	1.2
Local major roads (ROADS)	0 to year 8	0.86	0.21	0.34	0.62	0.86	1.03	1.44	3.67	0.41	0.36
	Year 9 to 16	0.61	0.13	0.22	0.43	0.62	0.74	1.02	3.56	0.31	0.26
	0 to year 16	0.73	0.17	0.29	0.53	0.75	0.89	1.20	3.54	0.36	0.30
Local other sources (GRID)	0 to year 8	3.87	1.08	2.00	3.14	4.06	4.55	5.39	9.82	1.41	1.06
	Year 9 to 16	2.63	0.66	1.27	2.13	2.83	3.15	3.65	6.40	1.02	0.77
	0 to year 16	3.25	0.88	1.66	2.67	3.44	3.84	4.49	7.95	1.17	0.89

a The total may not be equal to the sum of other components (ROAD + GRID + constant) due to rounding.

Correlations (p < 0.001) between ST-PM10_TOTAL_ for trimesters, early infancy, and late infancy (Table S4) were mostly low or very low (r: −0.15–0.23) with the exception of T1 and early infancy (r = 0.45), and T2 and late infancy (r = 0.72). For the same periods, there was very low correlation (Table S5) between NAME and ROAD (r: 0.08–0.18), very low to low correlation between NAME and GRID (r: 0.07–0.28), but GRID and ROAD were highly correlated (r = 0.75). Exposures between the period up to the first follow-up clinic (years 1 to 8) and the period from the first to second follow-up clinic (years 9 to 16) were very highly correlated (p < 0.001) for ROAD (r = 0.88), GRID (r = 0.89) and LT-PM10_TOTAL_ (r = 0.91). ROAD and GRID exposures within these periods were also very highly correlated (r = 0.82) (Table S5).

## 4 Discussion

### 4.1 Summary

We conducted daily modelling of source-specific (major road, other local, regional/long-range) particulate exposure for the critical biological windows of pregnancy and early infancy in the early 1990s, and annual average exposure modelling thereafter to age ~15 years. In doing so we accounted for changes in residential address. We evaluated the data outputs carefully against the limited contemporary data from a single monitoring station in the study area. Our evaluation adds to a paucity of information in the open literature on the performance of models in predicting short-term exposures in the early 1990s. Exposure estimates will be used in a number of planned epidemiological studies. Data will be added to the ALSPAC resource for other users (http://www.bristol.ac.uk/alspac/researchers/).

### 4.2 Model performance

Our model estimates correlated well with weekly measured PM_10_ concentrations in Bristol for 1993, a period up to one year beyond the last pregnancy in ALSPAC and overlapping early/late infancy for some of the cohort. We expected the mean bias (21%) to be lower for longer averaging periods in our study (e.g. trimesters), but measurements of PM_10_ concentrations were not sufficient to confirm this. Few other studies have evaluated the performance of PM_10_ dispersion modelling for averaging periods of less than a year and we are not aware of any such studies for the 1990s. In Christchurch, New Zealand, dispersion modelling was used to model daily concentrations of PM_10_ over two winter months (July 2003 and June 2004) for 11 intra-urban sites (Wilson and Zawar-Reza, 2006). Absolute bias for the two-monthly averages was highly variable between sites (−33–44%) and mean bias for the 11 sites was 31%. Srimath et al. (2017) combined models of local (CAR-FMI) and background (UK emissions model) PM_10_ in London and found strong correlation (r^2^ = 0.68) between daily measured and predicted PM_10_ concentrations at a single roadside site for 2008. The modelling, however, under-estimated measured PM_10_ concentrations by ~20%. Other studies used distance from residence to the nearest major road and local models to estimate traffic-related exposures for some gaseous pollutants (e.g. NO_X_, NO_2_), but relied on average concentration values from one monitoring site within each community as the exposure for PM_2.5_ and PM_10_ (Heck et al., 2013; McConnell et al., 2010; Urman et al., 2014).

In general, model performance tends to be better for annual PM_10_ concentrations than it does for short-term PM_10_ concentrations (e.g. daily, weekly). Beevers et al. (2013), for example, using a combination of a local model based on ADMS-Urban (KCL-Urban) and background model (CMAQ) in London, reported very high correlation (r^2^ = 0.96) and almost zero bias between measured and modelled PM_10_ for 2008, albeit with a relatively low number of sites (n = 12). Model performance in other studies has been good except where there is insufficient granularity and/or coverage of local sources (Deutsch et al., 2008; de Hoogh et al., 2014). We were unable to quantify the spatial bias in long-term exposures in ALSPAC as our evaluation was limited to a single monitoring site at any time (1993–2008).

### 4.3 Exposure contrasts

PM_10_ is a complex pollutant having both primary and secondary contributions and a varied chemical composition and associated sources. The coarse component (between 2.5 and 10 μm) of PM_10_ will generally be dominated by local sources, since the long-range coarse contribution tends to sediment/deposit out during transport over longer distances. The majority of the fine component (size < 2.5 μm) of PM_10_ tends to be secondary aerosol, formed from both local and distant gas phase precursors and transported on a regional scale. A relatively small and direct (i.e. primary) contribution is from local sources. It follows that the largest contribution (up to 46% of ST-PM10_TOTAL_) to exposures during pregnancy and early/late infancy was from the NAME component (Fig. S4). In our study local road sources (ROAD) made on average the smallest contribution to exposures (~3%) and the contribution of local other sources (GRID) was on average greater than ROAD by a factor of >5 (~16% of total). In a mother-child (n = 1154) study of air pollution and birth weight in France (Rahmalia et al., 2012), local exposures modelled with ADMS-Urban represented (based on our interpretation of data presented in Table 1 of Rahmalia et al., 2012) about 15% and 12% of total PM_10_ exposures in Nancy and Poitiers, respectively.

Intra-subject exposure contrasts over longer averaging periods (e.g. from birth to age 8 years) are related only to spatial variations in local sources of PM_10_, accounting for residential mobility, and were small in this study. Similarly, other studies (Beevers et al., 2013; Carruthers et al., 2003) have shown that, although at some sites by main roads PM_10_ concentrations were >5 μg/m^3^ higher than those found in nearby background areas, at other main roads PM_10_ concentrations were only 1–2 μg/m^3^ higher than nearby urban background sites. Air pollution gradients can be steep in the wake of road sources, reaching background levels at a variable distance (e.g. 20–100 m) from source, which is dependent on the street configuration and land cover between source and address (e.g. built-up area versus open-country); we studied this by running ADMS-Urban under different wind conditions (direction and speed) and for a hypothetical main road of 500 m in length with a daily average traffic flow of 12,000 vehicles (40 km h^−1^), PM_10_ concentrations perpendicular to the road reduced on average by 4.8 μg/m^3^ from 10 m to 50 m and by 5.6 μg/m^3^ from 10 m to 100 m. Air pollution gradients are especially steep in the wake of some main roads. Differences of >10 μg/m^3^ for measurements of annual average PM_10_ have been shown between kerbside (within 1 m of the kerb) and roadside (2–10 m from the kerb) sites in the UK (Beevers et al., 2013; Carruthers et al., 2003). It is not surprising therefore that exposure contrasts between subjects in ALSPAC, most of whom do not live close to main roads (i.e. 9.5% of address locations are within 5–50 m of a main road), were relatively small.

Fecht et al. (2016) separately assessed the contribution of road traffic and other sources to annual average (2003–2010) PM_10_ exposures for 190,122 postcodes (average of 12 households per postcode) in the area of Greater London. PM_10_ exposures from road traffic varied, depending on the year, between 2.9 μg/m^3^ and 3.5 μg/m^3^. This equates to 11–15% of total PM_10_ exposures, which is a factor of 4–5 times higher than in our study in Bristol for an overlapping period (2003–2008). The Fecht et al. study was, however, conducted in a mega-city of >8 million residents, with a higher density of main roads (0.5 km/km^2^ in the ALSPAC study area compared to 3 km/km^2^ in Greater London), and 4 times the number (38.5%) of address locations within 50 m of a main road than in our study area. Even though our study and these studies are not directly comparable, due to different locations and time periods, we believe that exposure contrasts due to main roads that we have estimated in ALSPAC are thus generally of the correct magnitude.

We found low correlations between pregnancy trimester and infancy exposures, which has been seen in the small number of other studies with information on both pregnancy trimester and early life exposures. For PM_10_, Van den Hooven et al. (2012), in the Generation R study in The Netherlands, reported low to moderate correlation (r: 0.31–0.48) between trimesters and low correlation between early and late infancy (r = 0.21). We found moderate correlation between trimester 1 and early infancy and high correlation between trimester 2 and late infancy. These periods overlap seasonally but their timing is not a determinant of correlations per se, as elevated PM_10_ exposures in north-western Europe, which are often related to low wind speeds and easterly air flow, can happen throughout the year (Stedman, 2004). For epidemiological studies interested in the effects of separate PM_10_ source components, there was low correlation between NAME and local exposure components (ROAD + GRID), and high, but not very high, correlation between ROAD and GRID. Thus, although local sources make up a relatively small proportion of total PM_10_, these correlations indicate that local sources could have independence and be significant in epidemiological studies.

### 4.4 Limitations and strengths

Although we gathered a large amount of detailed information on local and non-local emissions sources and meteorological data to run the dispersion models, our study has a number of limitations. We did not have information on emissions from individual point sources (e.g. industrial stacks) as this was not available for the earlier years in the study period, so instead we used aggregated emissions information on all sources, except main roads, on a 1 km grid. This may mean that we have under- or over-estimated the contribution of individual sources to PM_10_ concentrations. We were limited to using national-scale time-varying emissions factors as there is no information available on local factors to cover the study period. Furthermore, we did not include emissions of SO_2_ within the study area meaning that sulphate chemistry (i.e. the creation of secondary particulates by oxidising SO_2_ to form ammonium sulphate) was not accounted for in modelling PM_10_ with ADMS-Urban. This may have contributed to an underestimation of local sources of PM_10_ concentrations in some locations. In turn, this may also mean that our values of background concentrations are too large as they were determined by subtracting the sum of model estimates from measured concentrations of PM_10_.

We modelled dispersion of local sources of PM_10_ without accounting for the effects of terrain or buildings. There are options to include terrain and buildings in ADMS-Urban, but it was not practicable to model building effects over a large area as only 30 buildings could be included in a single model run of the version of ADMS-Urban that we used. We did not model the effects of terrain as this would have substantially increased the run time of our models beyond what was practicable. We recognize that although the study area is relatively flat, not including terrain in some locations may have contributed to errors in our exposure estimates. We did not have enough information on rainfall to model wet deposition with ADMS-Urban, but we believe this did not greatly affect our exposure estimates as we averaged over periods of at least a week for model evaluation and at least a trimester when assigning exposures.

We applied a single constant of 12 μg/m^3^ that was imputed from limited (1993) measured PM_10_ concentrations in the study area, to represent local ‘natural’ sources in estimates of ST-PM10_TOTAL_. In reality this will vary over time depending on meteorology, but we are unable to account for this during the pregnancy and early life period as we needed exposure estimates for periods prior to 1993 when there was no PM_10_ monitoring within the study area. A value of 12.0 μg/m^3^ is approximately the 1st centile of measured PM_10_ concentrations from 1993 at the Bristol Centre site; thus we believe it to be of the correct magnitude to represent an average PM_10_ concentration without the presence of local anthropogenic source. We imputed yearly constants from two monitoring sites in the centre of Bristol to represent ‘background’ PM_10_ (i.e. natural sources and regional/long-range aerosol from outside the study area). These may contribute to over-estimating background LT-PM_10_ concentrations for other locations, but there was no other monitoring of PM_10_ in the study area at any time. The nearest rural site (Narberth, Wales) was operational from 1997, but being 140 km to the west raises doubts about its representativeness of background concentrations for Avon.

Despite these limitations we have produced detailed exposure models from conception through to age 15 years (1990–2008) for different critical life periods, using a methodology where we are able to separately quantify local and non-local sources of PM_10_ for epidemiological investigations. It is a strength of the study that we were able to undertake model evaluation for exposure periods overlapping with pregnancy and early life in the early 1990s. A further strength is that we were able to account for residential mobility over the whole study period for a large proportion of ALSPAC participants.

## Funding

The research was supported by The UK Medical Research Council/Wellcome Trust (Grant ref.: 102215/2/13/2), the University of Bristol provide core support for ALSPAC. The work of the UK Small Area Health Statistics Unit is funded by Public Health England as part of the MRC-PHE Centre for Environment and Health, funded also by the UK Medical Research Council (Grant ref.: MR/L01341X/1). This paper does not necessarily reflect the views of Public Health England or the Department of Health.

## Appendix A Supplementary data

Supplementary material Image 1

## References

[bb0005] Atkinson R.W., Carey I.M., Kent A.J., van Staa T.P., Anderson H.R., Cook D.G. (2015). Long-term exposure to outdoor air pollution and the incidence of chronic obstructive pulmonary disease in a national English cohort. Occup. Environ. Med..

[bb0010] Baiz N., Dargent-Molina P., Wark J.D., Souberbielle J.C., Slama R., Annesi-Maesano I., EDEN Mother-Child Cohort Study Group (2012). Gestational exposure to urban air pollution related to a decrease in cord blood vitamin d levels. J. Clin. Endocrinol. Metab..

[bb0015] Beevers S.D., Kitwiroon N., Williams M.L., Kelly F.J., Ross Anderson H., Carslaw D.C. (2013). Air pollution dispersion models for human exposure predictions in London. J. Expo. Sci. Environ. Epidemiol..

[bb0020] Bellander T., Berglind N., Gustavsson P., Jonson T., Nyberg F., Pershagen G., Jarup L. (2001). Using geographic information systems to assess individual historical exposure to air pollution from traffic and house heating in Stockholm. Environ. Health Perspect..

[bb0025] Bennett N.D., Croke B.F.W., Guariso G., Guillaume J.H.A., Hamilton S.H., Jakeman A.J., Marsili-Libelli S., Newham L.T.H., Norton J.P., Perrin C., Pierce S.A., Robson B., Seppelt R., Voinov A.A., Fath B.D., Andreassian V. (2013). Characterising performance of environmental models. Environ. Model. Softw..

[bb0030] Berrisford P. (2011). The ERA-Interim archive version 2.0. ERA Report Series 1.

[bb0035] Boyd A., Golding J., Macleod J., Lawlor D.A., Fraser A., Henderson J., Molloy L., Ness A., Ring S., Davey Smith G. (2013). Cohort Profile: the ‘children of the 90s’—the index offspring of the Avon Longitudinal Study of Parents and Children. Int. J. Epidemiol..

[bb0040] Brunst K.J., Ryan P.H., Brokamp C., Bernstein D., Reponen T., Lockey J., Khurana Hershey G.K., Levin L., Grinshpun S.A., LeMasters G. (2015). Timing and duration of traffic-related air pollution exposure and the risk for childhood wheeze and asthma. Am. J. Respir. Crit. Care Med..

[bb0045] Bush T., Smith S., Stevenson K., Moorcroft S. (2001). Validation of nitrogen dioxide diffusion tube methodology in the UK. Atmos. Environ..

[bb0050] Butland B.K., Atkinson R.W., Crichton S., Barratt B., Beevers S., Spiridou A., Hoang U., Kelly F.J., Wolfe C.D. (2017). Air pollution and the incidence of ischaemic and haemorrhagic stroke in the South London Stroke Register: a case-cross-over analysis. J. Epidemiol. Community Health.

[bb0055] Carruthers D.J., Holroyd R.J., Hunt J.C.R., Weng W.-S., Robins A.G., Apsley D.D., Thompson D.J., Smith F.B. (1994). UK-ADMS-Urban: a new approach to modelling dispersion in the earth's atmospheric boundary layer. J. Wind Eng. Ind. Aerodyn..

[bb0060] Carruthers D.J., Blair K., Johnson K. (2003). Validation and sensitivity of ADMS-Urban-Urban for London Cambridge Environmental Research Consultants: TR0191. https://uk-air.defra.gov.uk/assets/documents/reports/cat09/Validation&Sensitivity(22JAN03)10_TR-0191-h.pdf.

[bb0065] Charpin D., Penard-Morand C., Raherison C., Kopferschmitt C., Lavaud F., Caillaud D., Annesi-Maesano I. (2009). Long-term exposure to urban air pollution measured through a dispersion model and the risk of asthma and allergy in children. Bull. Acad. Natl Med..

[bb0070] Deutsch F., Mensink C., Vankerkom J., Janssen L. (2008). Application and validation of a comprehensive model for PM10 and PM2.5 concentrations in Belgium and Europe. Appl. Math. Model..

[bb0075] Fecht D., Hansell A.L., Morley D., Dajnak D., Vienneau D., Beevers S., Toledano M.B., Kelly F.J., Anderson H.R., Gulliver J. (2016). Spatial and temporal associations of road traffic noise and air pollution in London: implications for epidemiological studies. Environ. Int..

[bb0080] Fuertes E., Standl M., Cyrys J., Berdel D., von Berg A., Bauer C.P., Kramer U., Sugiri D., Lehmann I., Koletzko S., Carlsten C., Brauer M., Heinrich J. (2013). A longitudinal analysis of associations between traffic-related air pollution with asthma, allergies and sensitization in the GINIplus and LISAplus birth cohorts. PeerJ.

[bb0085] Fuertes E., Bracher J., Flexeder C., Markevych I., Klumper C., Hoffmann B., Kramer U., von Berg A., Bauer C.P., Koletzko S., Berdel D., Heinrich J., Schulz H. (2015). Long-term air pollution exposure and lung function in 15 year-old adolescents living in an urban and rural area in Germany: the GINIplus and LISAplus cohorts. Int. J. Hyg. Environ. Health.

[bb0090] Wilson J.G., Zawar-Reza P. (2006). Intraurban-scale dispersion modelling of particulate matter concentrations: applications for exposure estimates in cohort studies. Atmos. Environ..

[bb0095] Gauderman W.J., Urman R., Avol E., Berhane K., McConnell R., Rappaport E., Chang R., Lurmann F., Gilliland F. (2015). Association of improved air quality with lung development in children. N. Engl. J. Med..

[bb0100] Gehring U., Wijga A.H., Hoek G., Bellander T., Berdel D., Bruske I., Fuertes E., Gruzieva O., Heinrich J., Hoffmann B., de Jongste J.C., Klumper C., Koppelman G.H., Korek M., Kramer U., Maier D., Melen E., Pershagen G., Postma D.S., Standl M., von Berg A., Anto J.M., Bousquet J., Keil T., Smit H.A., Brunekreef B. (2015). Exposure to air pollution and development of asthma and rhinoconjunctivitis throughout childhood and adolescence: a population-based birth cohort study. Lancet Respir. Med..

[bb0105] Gong T., Dalman C., Wicks S., Dal H., Magnusson C., Lundholm C., Almqvist C., Pershagen G. (2017). Perinatal exposure to traffic-related air pollution and autism spectrum disorders. Environ. Health Perspect..

[bb0110] Hampel R., Lepeule J., Schneider A., Bottagisi S., Charles M.A., Ducimetiere P., Peters A., Slama R. (2011). Short-term impact of ambient air pollution and air temperature on blood pressure among pregnant women. Epidemiology.

[bb0115] Hansen A.B., Ravnskjaer L., Loft S., Andersen K.K., Brauner E.V., Baastrup R., Yao C., Ketzel M., Becker T., Brandt J., Hertel O., Andersen Z.J. (2016). Long-term exposure to fine particulate matter and incidence of diabetes in the Danish Nurse Cohort. Environ. Int..

[bb0120] Heck J.E., Wu J., Lombardi C., Qiu J., Meyers T.J., Wilhelm M., Cockburn M., Ritz B. (2013). Childhood cancer and traffic-related air pollution exposure in pregnancy and early life. Environ. Health Perspect..

[bb0125] de Hoogh K., Korek M., Vienneau D., Keuken M., Kukkonen J., Nieuwenhuijsen M.J., Badaloni C., Beelen R., Bolignano A., Cesaroni G., Pradas M.C., Cyrys J., Douros J., Eeftens M., Forastiere F., Forsberg B., Fuks K., Gehring U., Gryparis A., Gulliver J., Hansell A.L., Hoffmann B., Johansson C., Jonkers S., Kangas L., Katsouyanni K., Kunzli N., Lanki T., Memmesheimer M., Moussiopoulos N., Modig L., Pershagen G., Probst-Hensch N., Schindler C., Schikowski T., Sugiri D., Teixido O., Tsai M.Y., Yli-Tuomi T., Brunekreef B., Hoek G., Bellander T. (2014). Comparing land use regression and dispersion modelling to assess residential exposure to ambient air pollution for epidemiological studies. Environ. Int..

[bb0130] Hsu H.H., Chiu Y.H., Coull B.A., Kloog I., Schwartz J., Lee A., Wright R.O., Wright R.J. (2015). Prenatal particulate air pollution and asthma onset in urban children. Identifying sensitive windows and sex differences. Am. J. Respir. Crit. Care Med..

[bb0135] Jones A.R., Thomson D.J., Hort M., Devenish B., Borrego C., Norman A.-L. (2007). The U.K. Met Office's next-generation atmospheric dispersion model, NAME III. Air Pollution Modeling and Its Application XVII (Proceedings of the 27th NATO/CCMS International Technical Meeting on Air Pollution Modelling and Its Application).

[bb0140] Keuken M., Zandveld P., van den Elshout S., Janssen N.A.H., Hoek G. (2011). Air quality and health impact of PM10 and EC in the city of Rotterdam, the Netherlands in 1985–2008. Atmos. Environ..

[bb0145] Korek M.J., Bellander T.D., Lind T., Bottai M., Eneroth K.M., Caracciolo B., de Faire U.H., Fratiglioni L., Hilding A., Leander K., Magnusson P.K., Pedersen N.L., Ostenson C.G., Pershagen G., Penell J.C. (2015). Traffic-related air pollution exposure and incidence of stroke in four cohorts from Stockholm. J. Expo. Sci. Environ. Epidemiol..

[bb0150] McConnell R., Islam T., Shankardass K., Jerrett M., Lurmann F., Gilliland F., Gauderman J., Avol E., Kunzli N., Yao L., Peters J., Berhane K. (2010). Childhood incident asthma and traffic-related air pollution at home and school. Environ. Health Perspect..

[bb0155] Molter A., Simpson A., Berdel D., Brunekreef B., Custovic A., Cyrys J., de Jongste J., de Vocht F., Fuertes E., Gehring U., Gruzieva O., Heinrich J., Hoek G., Hoffmann B., Klumper C., Korek M., Kuhlbusch T.A., Lindley S., Postma D., Tischer C., Wijga A., Pershagen G., Agius R. (2015). A multicentre study of air pollution exposure and childhood asthma prevalence: the ESCAPE project. Eur. Respir. J..

[bb0160] Morales E., Garcia-Esteban R., de la Cruz O.A., Basterrechea M., Lertxundi A., de Dicastillo M.D., Zabaleta C., Sunyer J. (2015). Intrauterine and early postnatal exposure to outdoor air pollution and lung function at preschool age. Thorax.

[bb0165] Mortimer K., Neugebauer R., Lurmann F., Alcorn S., Balmes J., Tager I. (2008). Air pollution and pulmonary function in asthmatic children: effects of prenatal and lifetime exposures. Epidemiology.

[bb0170] Nordling E., Berglind N., Melen E., Emenius G., Hallberg J., Nyberg F., Pershagen G., Svartengren M., Wickman M., Bellander T. (2008). Traffic-related air pollution and childhood respiratory symptoms, function and allergies. Epidemiology.

[bb0175] Pirani M., Gulliver J., Fuller G.W., Blangiardo M. (2014). Bayesian spatiotemporal modelling for the assessment of short-term exposure to particle pollution in urban areas. J. Expo. Sci. Environ. Epidemiol..

[bb0180] Rahmalia A., Giorgis-Allemand L., Lepeule J., Philippat C., Galineau J., Hulin A., Charles M.A., Slama R., EDEN Mother-Child Cohort Study group (2012). Pregnancy exposure to atmospheric pollutants and placental weight: an approach relying on a dispersion model. Environ. Int..

[bb0185] Rice M.B., Rifas-Shiman S.L., Litonjua A.A., Oken E., Gillman M.W., Kloog I., Luttmann-Gibson H., Zanobetti A., Coull B.A., Schwartz J., Koutrakis P., Mittleman M.A., Gold D.R. (2016). Lifetime exposure to ambient pollution and lung function in children. Am. J. Respir. Crit. Care Med..

[bb0190] Sbihi H., Tamburic L., Koehoorn M., Brauer M. (2016). Perinatal air pollution exposure and development of asthma from birth to age 10 years. Eur. Respir. J..

[bb0195] Schultz E.S., Hallberg J., Pershagen G., Melen E. (2016). Reply: early-life exposure to traffic-related air pollution and lung function in adolescence. Am. J. Respir. Crit. Care Med..

[bb0200] Sellier Y., Galineau J., Hulin A., Caini F., Marquis N., Navel V., Bottagisi S., Giorgis-Allemand L., Jacquier C., Slama R., Lepeule J. (2014). Health effects of ambient air pollution: do different methods for estimating exposure lead to different results?. Environ. Int..

[bb0205] Srimath S.T.G., Sokhi R., Karppinen A., Singh V., Kukkonen J. (2017). Evaluation of an urban modelling system against three measurement campaigns in London and Birmingham. Atmos. Pollut. Res..

[bb0210] Stedman J.R. (2004). The predicted number of air pollution related deaths in the UK during the August 2003 heatwave. Atmos. Environ..

[bb0215] Urman R., McConnell R., Islam T., Avol E.L., Lurmann F.W., Vora H., Linn W.S., Rappaport E.B., Gilliland F.D., Gauderman W.J. (2014). Associations of children's lung function with ambient air pollution: joint effects of regional and near-roadway pollutants. Thorax.

[bb0220] Van den Hooven E.H., Pierik F.H., Van Ratingen S.W., Zandveld P.Y., Meijer E.W., Hofman A., Miedema H.M., Jaddoe V.W., De Kluizenaar Y. (2012). Air pollution exposure estimation using dispersion modelling and continuous monitoring data in a prospective birth cohort study in The Netherlands. Environ. Health.

[bb0225] Vinceti M., Malagoli C., Malavolti M., Cherubini A., Maffeis G., Rodolfi R., Heck J.E., Astolfi G., Calzolari E., Nicolini F. (2016). Does maternal exposure to benzene and PM10 during pregnancy increase the risk of congenital anomalies? A population-based case-control study. Sci. Total Environ..

