# Supplementary material for: Local- and regional-scale air pollution modelling (PM10) and exposure assessment for pregnancy trimesters, infancy, and childhood to age 15 years: Avon Longitudinal Study of Parents And Children (ALSPAC)
Source: Environ Int. 2018 Apr;113:10–9. doi: 10.1016/j.envint.2018.01.017 (PMC5907299; doi:10.1016/j.envint.2018.01.017)
Supplement: Supplementary file 1 — Supplementary material [file mmc1.docx]

**SUPPORTING INFORMATION**

| Contents | Page |
| --- | --- |
|  |  |
| Figure S1. Annual UK PM_10_ emissions (kilo tonnes) by source sector from 1990 to 2009. | 2 |
| Figure S2. Annual and monthly average measured PM_10_ concentrations from Bristol monitoring sites (1993-2008). | 3 |
| Figure S3. Modelled (ST-PM10_TOTAL_) versus measured PM_10_ (μg/m^3^) for 37 weeks during 1993 at the Bristol Centre monitoring site. | 4 |
| Figure S4. Proportional contribution of ROAD, GRID, NAME, and NATURAL (a constant value of 12 μg/m^3^) to ST-PM10_TOTAL_ (μg/m^3^) for the 37 weeks in 1993 where measurements of PM_10_ (μg/m^3^) were available from the ‘Bristol Centre’ monitoring site. | 5 |
| Figure S5. Distributions of mean PM_10_ (μg/m^3^) exposures for different life periods relating to follow-up clinics: A) birth to year 8; B) year 9 to year 16, C) birth to year 16. Total N in each case = 10383. | 6 |
| Table S1. Yearly model constants in the calculation of LT-PM10_TOTAL_ (i.e. ROAD + GRID + BACKGROUND). | 7 |
| Table S2. Number of individuals with valid address records for pregnancy trimesters (T1, T2, T3), early infancy (EI, months 1 to 6), late infancy (LI, months 7-12), and each year of life from birth up to age ~15 (Year 1, Year 2,……, Year 16). | 8 |
|  |  |
| Table S3. Long-term total PM_10_ exposures (μg/m^3^) for each year of life for individuals with complete address history in all periods (n = 10,383). | 9 |
| Table S4. Spearman’s correlation (rho.) between average modelled ST-PM10_TOTAL_ exposures for pregnancy trimesters, early infancy (months 1 to 6), and late infancy (months 7-12) (n = 11,929). | 10 |
| Table S5. Spearman’s correlation (rho.) between modelled ROAD, GRID, NAME, and ST-PM10_TOTAL_ by pregnancy trimester (T1, T2, T3), early infancy (EI; months 1 to 6), and late infancy (LI; months 7-12) (n = 11,929). | 11 |
| Table S6. Spearman’s correlation (rho) between modelled ROAD, GRID, and LT-PM10_TOTAL_ exposures for birth to year 8 and year 9 to 16 (n = 10,383). | 12 |
|  |  |

Figure S1. Annual UK PM_10_ emissions (kilo tonnes) by source sector from 1990 to 2009 (http://naei.beis.gov.uk/data/data-selector).


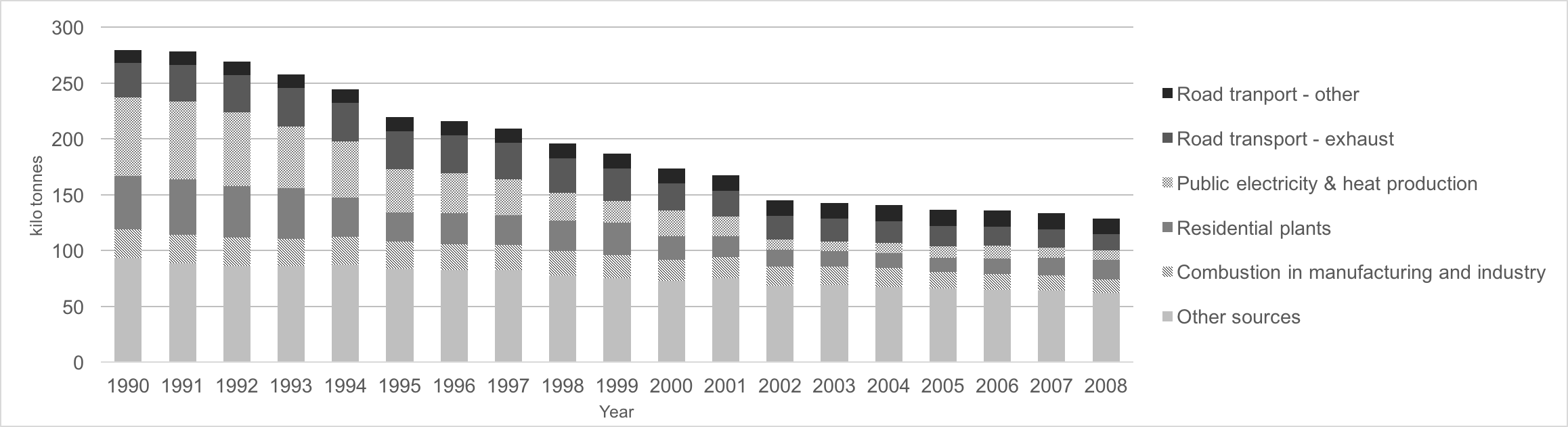


Figure S2. Annual and monthly average measured PM_10_ concentrations from Bristol monitoring sites (1993-2008).


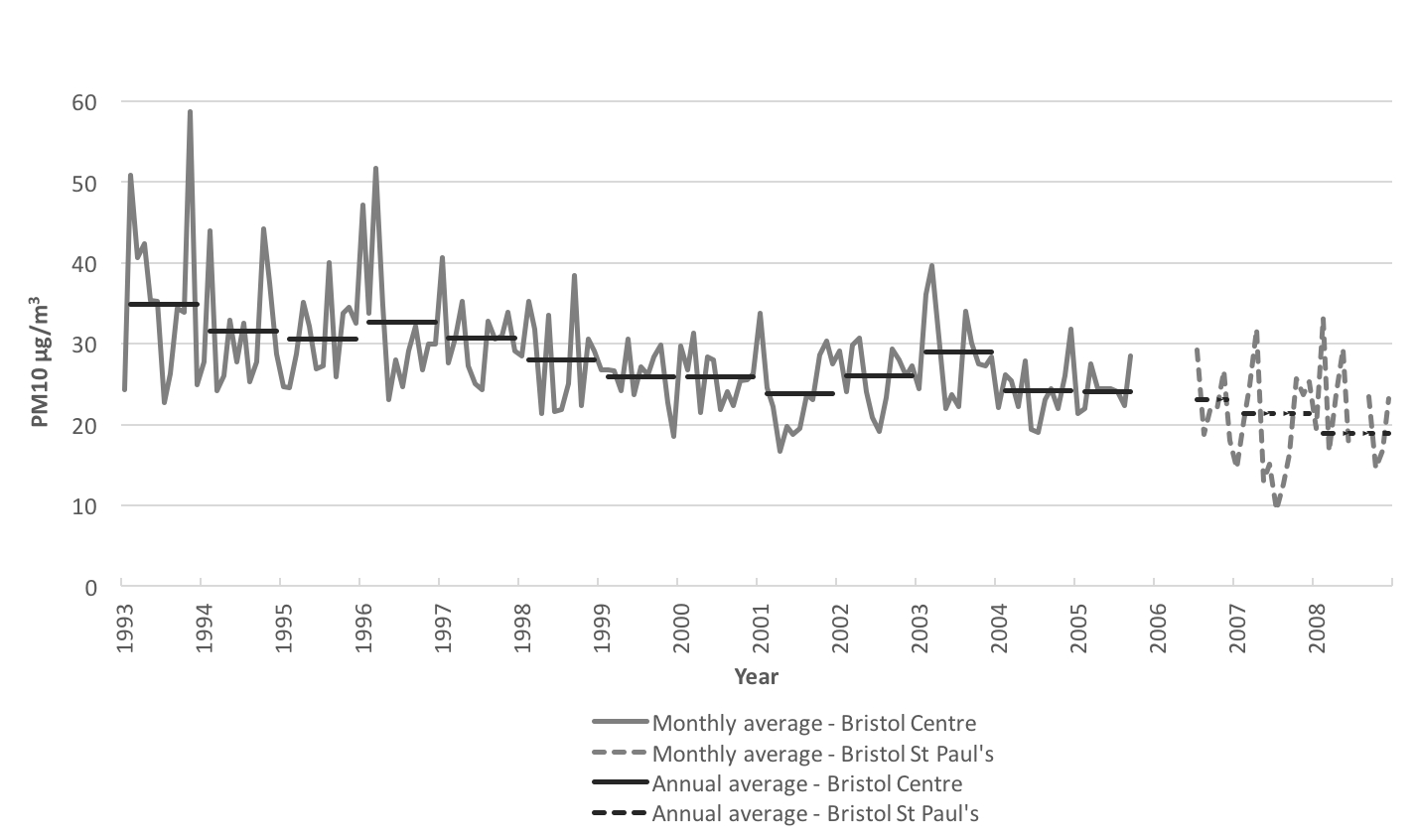


Figure S3. Modelled (ST-PM10_TOTAL_) versus measured PM_10_ (μg/m^3^) for 37 weeks during 1993 at the Bristol Centre monitoring site. The solid line is the model fit and unity (1:1) is shown by the dashed line.


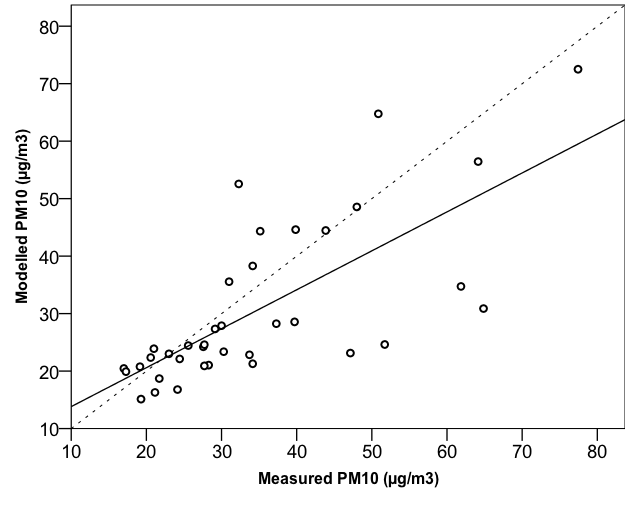


Figure S4. Proportional contribution of ROAD, GRID, NAME, and NATURAL (a constant value of 12 μg/m^3^) to ST-PM10_TOTAL_ (μg/m^3^) for the 37 weeks in 1993 where measurements of PM_10_ (μg/m^3^) were available from the ‘Bristol Centre’ monitoring site.


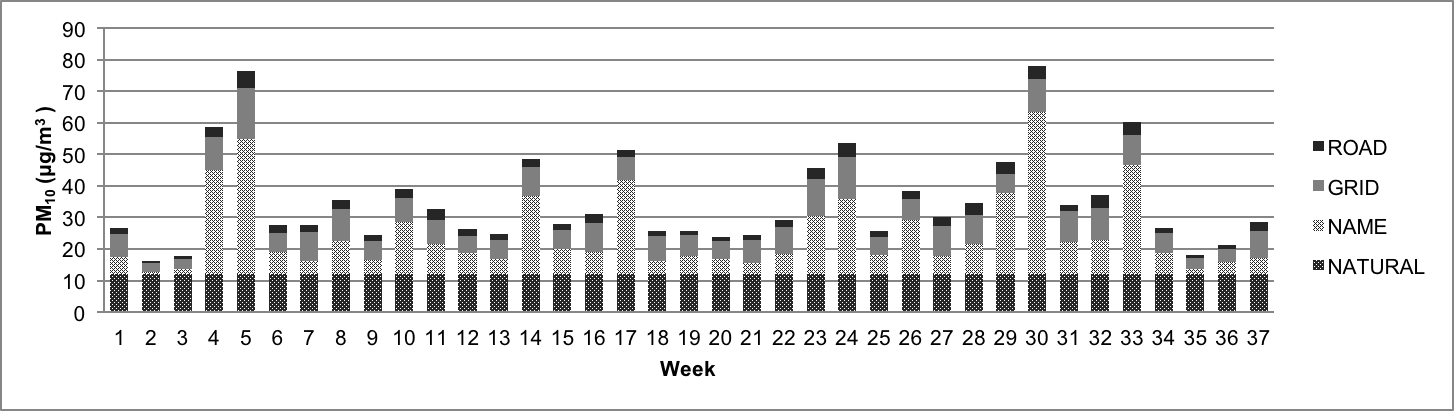


Figure S5. Distributions of mean PM_10_ (μg/m^3^) exposures for different life periods relating to follow-up clinics: A) birth to year 8; B) year 9 to year 16, C) birth to year 16. Total N in each case = 10383.


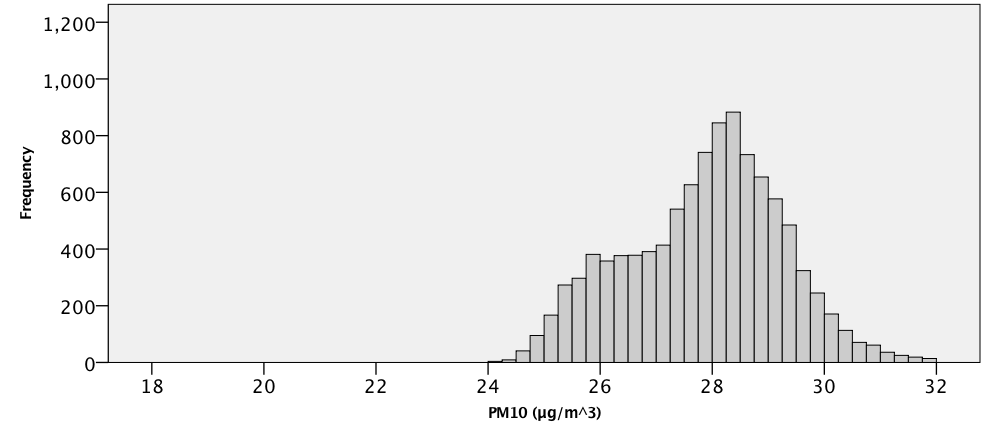


A


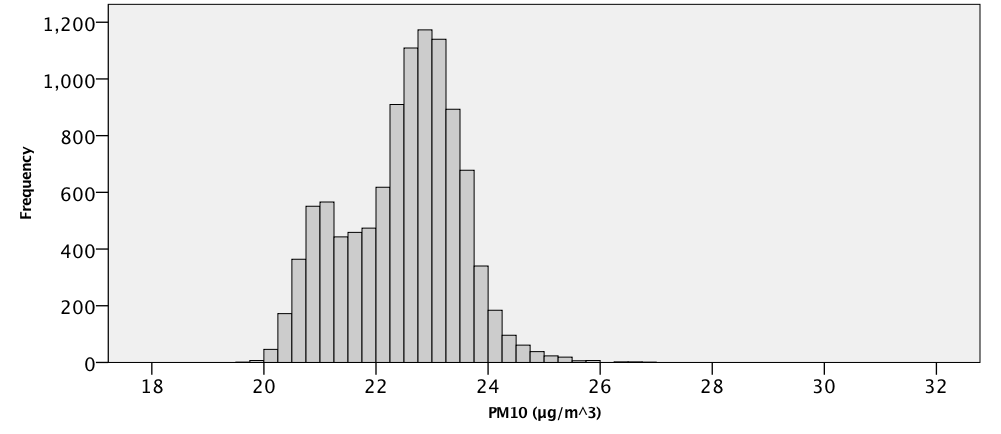


B


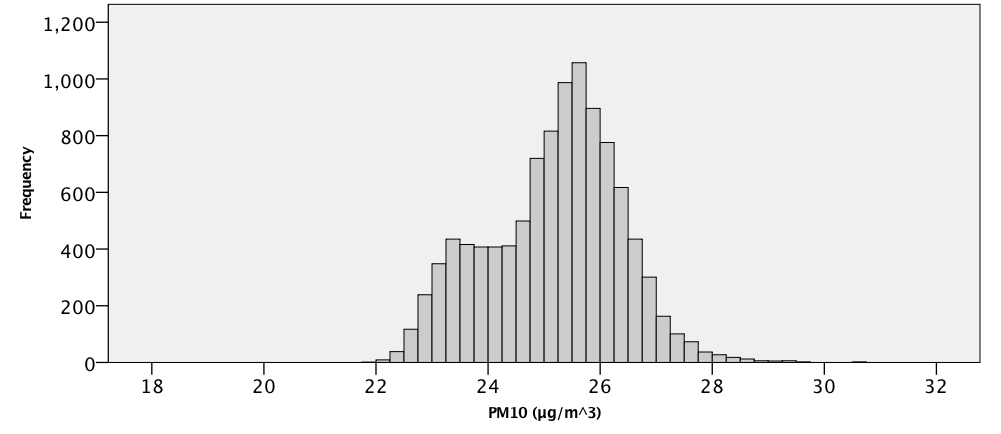


C

Table S1. Yearly model constants in the calculation of LT-PM10_TOTAL_ (i.e. ROAD + GRID + BACKGROUND). BACKGROUND is the difference between the sum of modelled ROAD and GRID and measured concentrations of PM_10_ (μg/m^3^) at Bristol monitoring sites (‘Centre’ or ‘St Paul’s’; Figure 1).

| Year | Monitoring site | Measured PM_10_ (μg/m^3^) | ROAD | GRID | BACKGROUND |
| --- | --- | --- | --- | --- | --- |
| 1990 | Centre | - | - | - | 24.3^1^ |
| 1991 | Centre | - | - | - | 24.3^1^ |
| 1992 | Centre | - | - | - | 24.3^1^ |
| 1993 | Centre | 34.9 | 2.58 | 7.99 | 24.3 |
| 1994 | Centre | 31.5 | 1.56 | 4.94 | 25.0 |
| 1995 | Centre | 30.6 | 1.94 | 5.67 | 23.0 |
| 1996 | Centre | 32.6 | 2.00 | 5.43 | 25.2 |
| 1997 | Centre | 30.7 | 2.21 | 5.91 | 22.6 |
| 1998 | Centre | 28.0 | 1.76 | 4.52 | 21.8 |
| 1999 | Centre | 25.9 | 1.83 | 4.49 | 19.6 |
| 2000 | Centre | 26.0 | 1.91 | 4.15 | 19.9 |
| 2001 | Centre | 23.8 | 1.34 | 4.30 | 18.2 |
| 2002 | Centre | 26.0 | 1.32 | 3.63 | 21.1 |
| 2003 | Centre | 29.0 | 1.41 | 3.77 | 23.8 |
| 2004 | Centre | 24.1 | 1.44 | 4.37 | 18.3 |
| 2005 | Centre | 24.1 | 1.47 | 4.61 | 18.0 |
| 2006 | St Paul’s | 23.1 | 0.84 | 3.81 | 18.5 |
| 2007 | St Paul’s | 21.4 | 0.82 | 3.81 | 16.8 |
| 2008 | St Paul’s | 18.9 | 0.52 | 2.22 | 16.2 |

^1^assumed to be the same value as 1993 as there were no PM_10_ measurements prior to 1993.

Table S2. Number of individuals with valid address records for pregnancy trimesters (T1, T2, T3), early infancy (EI, months 1 to 6), late infancy (LI, months 7-12), and each year of life from birth up to age ~15 (Year 1, Year 2,……, Year 16).

| Period | Valid records in each period | Valid records in all periods |
| --- | --- | --- |
| *Pregnancy and infancy* | | |
| T1 | 12494 | 11929 |
| T2 | 12775 |  |
| T3 | 12710 |  |
| EI | 12586 |  |
| LI | 12488 |  |
|  | | |
| *Yearly from birth to age 15* | | |
| Year 1 | 12503 | 10383 |
| Year 2 | 12355 |  |
| Year 3 | 12240 |  |
| Year 4 | 12160 |  |
| Year 5 | 12077 |  |
| Year 6 | 12003 |  |
| Year 7 | 11937 |  |
| Year 8 | 11822 |  |
| Year 9 | 11736 |  |
| Year 10 | 11661 |  |
| Year 11 | 11595 |  |
| Year 12 | 11549 |  |
| Year 13 | 11518 |  |
| Year 14 | 11503 |  |
| Year 15 | 11474 |  |
| Year 16 | 11446 |  |

Table S3. Long-term total PM_10_ exposures (μg/m^3^) for each year of life for individuals with complete address history in all periods (n = 10,383).

| Exposure Period | Mean | Min | Percentiles | | | | | Max | IQR | SD |
| --- | --- | --- | --- | --- | --- | --- | --- | --- | --- | --- |
|  |  |  | 5th | 25th | 50th | 75th | 95th |  |  |  |
| Year 1 | 31.2 | 26.3 | 27.8 | 29.7 | 31.4 | 32.5 | 34.3 | 40.5 | 2.7 | 2.0 |
| Year 2 | 30.1 | 25.9 | 27.3 | 28.7 | 30.1 | 31.2 | 33.0 | 39.2 | 2.5 | 1.8 |
| Year 3 | 28.6 | 24.5 | 26.3 | 27.6 | 28.7 | 29.5 | 31.0 | 37.2 | 1.9 | 1.4 |
| Year 4 | 28.3 | 24.7 | 25.9 | 27.4 | 28.5 | 29.3 | 30.4 | 33.7 | 1.9 | 1.4 |
| Year 5 | 28.7 | 24.5 | 26.4 | 27.7 | 28.9 | 29.7 | 30.7 | 33.6 | 2.0 | 1.4 |
| Year 6 | 27.2 | 22.9 | 24.5 | 26.2 | 27.2 | 28.3 | 29.7 | 33.9 | 2.2 | 1.6 |
| Year 7 | 25.2 | 20.7 | 22.5 | 24.2 | 25.2 | 26.3 | 27.6 | 31.8 | 2.1 | 1.6 |
| Year 8 | 23.7 | 20.4 | 21.4 | 22.9 | 23.9 | 24.5 | 25.7 | 29.1 | 1.6 | 1.3 |
| Year 9 | 22.9 | 19.4 | 20.7 | 22.0 | 23.0 | 23.9 | 24.7 | 28.2 | 1.9 | 1.3 |
| Year 10 | 22.5 | 19.5 | 20.5 | 21.7 | 22.6 | 23.3 | 24.4 | 26.9 | 1.6 | 1.2 |
| Year 11 | 24.4 | 19.9 | 21.8 | 23.3 | 24.4 | 25.6 | 27.0 | 29.8 | 2.3 | 1.6 |
| Year 12 | 25.0 | 19.5 | 22.0 | 23.9 | 25.2 | 26.2 | 27.2 | 31.0 | 2.3 | 1.6 |
| Year 13 | 22.5 | 18.9 | 19.9 | 21.6 | 22.5 | 23.4 | 25.3 | 29.1 | 1.8 | 1.6 |
| Year 14 | 21.7 | 19.0 | 19.8 | 21.0 | 22.0 | 22.5 | 23.2 | 26.2 | 1.5 | 1.1 |
| Year 15 | 21.2 | 17.9 | 19.2 | 20.3 | 21.3 | 22.0 | 22.9 | 25.9 | 1.7 | 1.1 |
| Year 16 | 19.6 | 16.8 | 17.7 | 18.6 | 19.5 | 20.6 | 21.8 | 24.8 | 2.0 | 1.3 |

Table S4. Spearman’s correlation (rho.) between average modelled ST-PM10_TOTAL_ exposures for pregnancy trimesters, early infancy (months 1 to 6), and late infancy (months 7-12) (n = 11,929). All correlations significant at the 0.01 level (2-tailed).

|  | Trimester 1 | Trimester 2 | Trimester 3 | EI 1-6m^a^ | LI 7-12m^b^ |
| --- | --- | --- | --- | --- | --- |
| Trimester 1 | 1 |  |  |  |  |
| Trimester 2 | -0.04 | 1 |  |  |  |
| Trimester 3 | -0.08 | 0.03 | 1 |  |  |
| EI 1-6m^a^ | 0.45 | -0.15 | 0.11 | 1 |  |
| LI 7-12m^b^ | 0.10 | 0.72 | 0.23 | 0.04 | 1 |

All correlations two-tailed; p<0.001.

^a^EI (1-6m): early infancy from birth to age 6 months; ^b^LI (7-12m): late infancy from 7-12 months

Table S5. Spearman’s correlation (rho.) between modelled ROAD, GRID, NAME, and ST-PM10_TOTAL_ by pregnancy trimester (T1, T2, T3), early infancy (EI; months 1 to 6), and late infancy (LI; months 7-12) (n = 11,929). All correlations significant at the 0.01 level (2-tailed).

|  | ROAD | | GRID | | NAME | |
| --- | --- | --- | --- | --- | --- | --- |
| GRID | T1 | 0.75 |  |  |  |  |
|  | T2 | 0.75 |  |  |  |  |
|  | T3 | 0.75 |  |  |  |  |
|  | EI | 0.75 |  |  |  |  |
|  | LI | 0.75 |  |  |  |  |
| NAME | T1 | 0.14 | T1 | 0.24 |  |  |
|  | T2 | 0.15 | T2 | 0.25 |  |  |
|  | T3 | 0.18 | T3 | 0.28 |  |  |
|  | EI | 0.13 | EI | 0.18 |  |  |
|  | LI | 0.08 | LI | 0.07 |  |  |
| ST-PM10_TOTAL_ | T1 | 0.47 | T1 | 0.60 | T1 | 0.90 |
|  | T2 | 0.48 | T2 | 0.61 | T2 | 0.89 |
|  | T3 | 0.54 | T3 | 0.68 | T3 | 0.86 |
|  | EI | 0.58 | EI | 0.68 | EI | 0.80 |
|  | LI | 0.63 | LI | 0.70 | LI | 0.72 |

Table S6. Spearman’s correlation (rho.) between modelled ROAD, GRID, and LT-PM10_TOTAL_ exposures for birth to year 8 and year 9 to 16 (n = 10,383). All correlations significant at the 0.01 level (2-tailed).

|  | ROAD | | GRID | |
| --- | --- | --- | --- | --- |
| GRID | birth to year 8 | 0.82 |  | |
|  | year 9 to 16 | 0.82 |  | |
| LT-PM10_TOTAL_ | birth to year 8 | 0.97 | birth to year 8 | 0.86 |
|  | year 9 to 16 | 0.95 | year 9 to 16 | 0.89 |
